# Signal Detection of Potentially Drug-Induced Liver Injury in Children Using Electronic Health Records

**DOI:** 10.3389/fped.2020.00171

**Published:** 2020-04-16

**Authors:** Yuncui Yu, Xiaolu Nie, Ziyang Song, Yuefeng Xie, Xuan Zhang, Zhaoyang Du, Ran Wei, Duanfang Fan, Yiwei Liu, Qiuye Zhao, Xiaoxia Peng, Lulu Jia, Xiaoling Wang

**Affiliations:** ^1^Clinical Research Center, National Center for Children's Health, Beijing Children's Hospital, Capital Medical University, Beijing, China; ^2^Center for Clinical Epidemiology and Evidence-Based Medicine, National Center for Children's Health, Beijing Children's Hospital, Capital Medical University, Beijing, China; ^3^Department of Pharmacy, National Center for Children's Health, Beijing Children's Hospital, Capital Medical University, Beijing, China; ^4^Information Center, National Center for Children's Health, Beijing Children's Hospital, Capital Medical University, Beijing, China; ^5^Department of Public Health and Preventive Medicine, School of Medicine, Keio University, Tokyo, Japan; ^6^Center of Big Data in Medicine, Beijing Institute of Big Data Research, Beijing, China

**Keywords:** post-marketing surveillance, drug safety, drug-induced liver injury, electronic health records, pediatrics

## Abstract

**Background:** This study proposes a quantitative 2-stage procedure to detect potential drug-induced liver injury (DILI) signals in pediatric inpatients using an data warehouse of electronic health records (EHRs).

**Methods:** Eight years of medical data from a constructed database were used. A two-stage procedure was adopted: (i) stage 1: the drugs suspected of inducing DILI were selected and (ii) stage 2: the associations between the drugs and DILI were identified in a retrospective cohort study.

**Results:** 1,196 drugs were filtered initially and 12 drugs were further potentially identified as suspect drugs inducing DILI. Eleven drugs (fluconazole, omeprazole, sulfamethoxazole, vancomycin, granulocyte colony-stimulating factor (G-CSF), acetaminophen, nifedipine, fusidine, oseltamivir, nystatin and meropenem) were showed to be associated with DILI. Of these, two drugs, nystatin [odds ratio[OR]=1.39, 95%CI:1.10–1.75] and G-CSF (OR = 1.91, 95%CI:1.55–2.35), were found to be new potential signals in adults and children. Three drugs [nifedipine [OR = 1.77, 95%CI:1.26–2.46], fusidine [OR = 1.43, 95%CI:1.08–1.86], and oseltamivi r [OR = 1.64, 95%CI:1.23–2.18]] were demonstrated to be new signals in pediatrics. The other drug-DILI associations had been confirmed in previous studies.

**Conclusions:** A quantitative algorithm to detect potential signals of DILI has been described. Our work promotes the application of EHR data in pharmacovigilance and provides candidate drugs for further causality assessment studies.

## Introduction

Drug-induced liver injury (DILI) is a serious public health issue and potentially serious adverse reaction that can acute liver failure. The incidence of DILI in developed countries is estimated to be 19/100,000 in the general population (1). Rates of DILI in inpatient wards are higher, ranging from 0.12 to 1.4 per 100 admissions (2). It accounts for 4–10% of all adverse drug reactions (ADR) and up to 13–15% of liver failure, with 29% of the liver failure cases had liver transplantation in American adults (3, 4). Recently, DILI has become the most important cause of post-marketing warnings and drug withdrawals (5). Moreover, children and adolescents, with a lack of clinical trials and immature liver and kidney function, are more prone to DILI than adults (6, 7). Thus, the detection of DILI signals is very important for post-marketing surveillance, especially in pediatric patients.

Traditionally, spontaneous reporting systems (SRSs), as the passive systems collecting reports of adverse drug events (ADEs), are the most common resources for monitoring DILI signals. However, these passive surveillance methods are limited by under-reporting, poor report quality, reporting bias, and unable to calculate the frequency of ADEs (8). A previous study showed that <6% of hepatic adverse reactions were reported (9). The expanding use of electronic health records (EHRs) during these years provides another potentially abundant source for pharmacovigilance and allows the use of larger populations, including children and adolescents, in population-based studies. These data are more practical and contribute to a more precise benefit-risk assessment.

Several studies have explored the signals of DILI in routinely collected data from EHRs, such as laboratory results and diagnosis codes (10–13). However, few studies focused on the drugs suspected of inducing DILI in children and adolescents. The study aims to conduct a two-stage algorithm with retrospective cohort designs to explore and evaluate potential DILI signals from large dataset of EHR. Thus, it can offer suspect drugs for pharmacovigilance and causality assessment researches of ADRs.

## Materials and Methods

### Dataset

A database containing the inpatients of Beijing Children's Hospital (BCH) was established previously, which including detailed visits, medications, clinical diagnosis as well as laboratory tests from January 1, 2010 to December 31, 2017. The database used in this study contained 379,160 hospital records from 247,136 patients aged 28 days to 18 years old, involving a total of 49,685,862 laboratory tests and 8,927,894 prescriptions. A hospitalization record represented one hospitalization, so there were multiple records if the same patients was hospitalized more than one time. In this study, all the data we used for eligible patients were exported from the data warehouse and deidentified to protect patients' privacy and confidentiality.

### Identification of Potentially DILI

Potentially DILI is mainly identified by temporary changes in laboratory chemical indicators related to drug use. According to the *Guidelines for Medical Nomenclature Use of Adverse Drug Reactions*, which was issued by the National Center for ADR Monitoring of the China Food and Drug Administration (CFDA) in 2016 (14), DILI was defined when the drug was administered within the therapeutic dose range, and the following events occurred within 90 days of initial administration: (1) any elevation of alanine aminotransferase (ALT) or total bilirubin (TB) greater than the upper limit of normal range (>ULN) in two successive tests; or (2) any elevation of ALT or TB greater than two times the ULN (>2 × ULN) in one test. The ULNs for the laboratory tests at the BCH were 40 IU/L and 20.5 mmol/L for ALT and TB, respectively. Alkaline phosphomonoesterase (ALP) was not chosen because increases in ALP activities in pediatric patients are mostly due to bones or other organs rather than liver disease (15).

#### Stage 1: Screening Drugs Potentially Causing DILI

**Figure 1:**
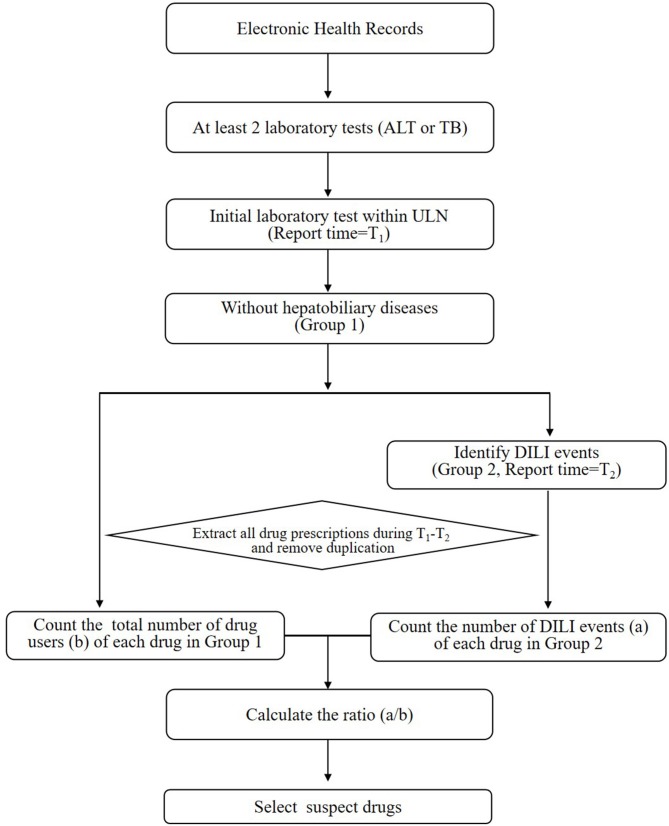
The workflow of stage 1 for screening drugs potentially causing DILI. ALT, alanine aminotransferase; ULN, upper limit of normal range; DILI, drug-induced liver injury; TB, total bilirubin.

The purpose of stage 1 was to identify potentially offending medications that deserved further research regarding their associations with DILI (shown in Figure 1). Only chemical medicine was involved in this study. When a patient used two or more drugs in one record, the record will be included in each drug's signal exploration, respectively. The main steps were as follows:

1. The hospital records that obtained at least two laboratory tests (ALT or TB) from admission to discharge were included;
2. The hospital records that obtained an initial ALT/TB results under the ULN and its report time (T1) were retained;
3. The hospital records containing a diagnosis of hepatobiliary disease (16) (shown in Table S1) that influenced the ALT or TB levels were excluded. The rest hospital records were defined as Group 1. This step was executed because the changes in ALT/TB levels of patients with hepatobiliary disease might be largely due to the progression of hepatobiliary disease itself, rather than DILI.
4. The hospital records of patients with DILI according to ALT and TB levels from Group 1 were included in Group 2.
5. The time of the first abnormal ALT/TB test were considered as T2. All drug prescriptions during the period from T1 to T2 in every record were collected. Duplicate prescription information was deleted.
6. The hospital records in Group 2 and Group1 was considered to be the number of drug adverse events (a) and the total number of drug users (b), respectively. Then ratio (a/b) for each drug was calculated.
7. The suspect drug that met following criteria were selected after expert consultation: (1) ratio (a/b)>0.15; (2) total users (b) >1,000. The a/b values of adjuvant drugs, such as normal saline and glucose injection, ranged from 0.09 to 0.11, which can be regarded as the value of background. And if b is too small, there may be a greater risk of bias when doing subsequent statistical analysis.

### Procedure Development

#### Stage 2: Identifying DILI Signals Based on Retrospective Cohort Designs

**Figure 2:**
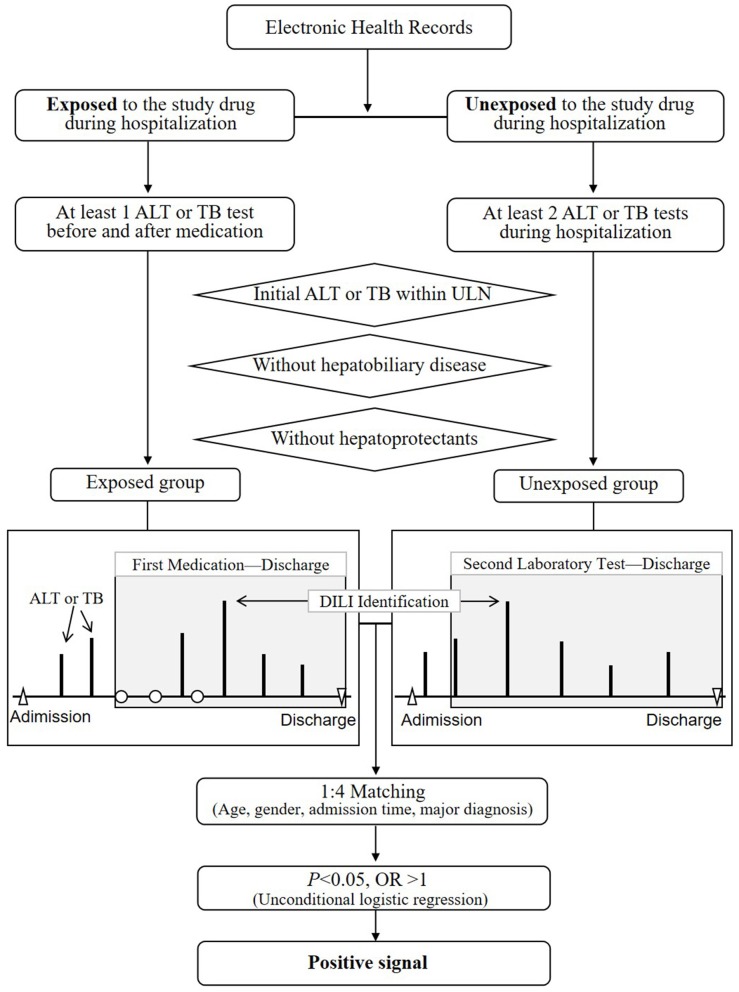
The overall design of stage 2 for the detection of DILI signals based on retrospective cohort designs. ALT, alanine aminotransferase; ULN, upper limit of normal range; DILI, drug-induced liver injury; OR, odds ratio; TB, total bilirubin.

The overall framework of stage 2 was displayed in Figure 2. The purpose of this step was to study the associations between drugs and DILI by comparing differences in DILI event rates between the exposed and unexposed group after adjusting for four confounders. According to retrospective cohort designs, every suspect drug filtered out in stage 1 was analyzed as follows:

**(1) Exposed group:**

1. The hospital records with the suspect drugs were included.
2. The hospital records that obtained at least two ALT or TB results before and after taking the suspect drug were included.
3. The hospital records that obtained the latest ALT or TB results within the ULNs before the first dose of medication were retained.
4. The hospital records that obtain diagnosed hepatobiliary diseases were also excluded (shown in Table S1).
5. For the rest records that obtained abnormal ALT or TB levels, the records which used the hepatoprotectants before the first report time of abnormal test were excluded (shown in Table S2). And for the rest records that did not obtained abnormal ALT or TB levels, the records which used the hepatoprotectants during the entire hospitalization were excluded.

**(2) Unexposed group:**

1. The hospital records without the suspect drugs were selected.
2. The hospital records that obtained at least two ALT or TB results from admission to discharge were identified.
3. The hospital records that obtained initial ALT or TB results within the ULNs were included.
4. The hospital records that obtained diagnosed hepatobiliary diseases were also excluded.
5. For the rest records that obtained abnormal ALT or TB levels, the records with hepatoprotectants before the time of the first abnormal ALT or TB levels were excluded. For the rest records that did not obtained abnormal ALT or TB levels, the records with the hepatoprotectants during hospitalization were excluded.

**(3) DILI signal detection**

1. Each exposed record was paired to four unexposed records randomly after adjusting age, gender, admission time, and major diagnosis (based on the classification in ICD-10).
2. The odds ratio (OR) and its 95% confidence interval (CI) was calculated using the unconditional logistic regression.
3. An OR>1.0 indicated a positive signal, otherwise a negative signals (OR ≤ 1).

#### Evaluation of the DILI Signals

The available knowledge from literature search as well as summary of product characteristics (SPCs) was used to evaluate the novelty of the DILI signals. The SPCs reviewed from micromedex (https://www.ibm.com/watson-health/learn/micromedex), FDA website (https://www.fda.gov) or drug instructions. Literature reviewed through PUBMED (https://pubmed.ncbi.nlm.nih.gov), Wanfang (http://www.wanfangdata.com.cn/index.html) as well as CNKI (http://www.cnki.net/).

### Software Tools Used and Statistics

Data management was performed by MySQL (Version 14.14). Statistical analysis was performed using R3.5.1 software. The GraphPad Prism 8.0.1 software was used to produce figures.

The possible confounding factors in exposed groups and unexposed groups were matched by propensity score matching (PSM) approach. Logistic regression model was used to calculate propensity scores, with drug exposure or not as dependent variables and four confounding factors (age, gender, admission time, and main diagnosis) as covariates. The nearest neighbor matching principle was used and matching ratio was set to 1: 4 in this process. The balance of covariates across the two groups in the matched sample was finally verified.

All *P*-values were reported two-sided. *P* < 0.05 represented statistical significance. The missing data was processed by the listwise deletion approach due to the low missing probabilities (<5%).

## Results

### Selection of Suspect Drugs

In stage 1, 1,196 drugs were filtered initially. After combining the same ingredient drugs with different dosages, specifications or manufacturers, 171 drugs remained. After excluding hepatoprotectants and adjuvant drugs, such as normal saline, 53 drugs were left. Among them, 12 drugs (fluconazole, omeprazole, sulfamethoxazole, vancomycin, phenobarbital, granulocyte colony-stimulating factor (G-CSF), acetaminophen, nifedipine, fusidine, oseltamivir, nystatin, and meropenem) met the inclusion criteria (b>1,000 and a/b>0.15). These twelve drugs were considered as suspect drugs and chosen for stage 2 with regard to DILI signals detection. More information was shown in Table 1.

**Table 1:** Information on the 12 suspect drugs.

**Drug ID**	**Suspect drugs**	**Number of DILI events (a)**	**Total number of drug users^a^(b)**	**Ratio (a/b)**
1	Fluconazole	904	3,405	0.27
2	Omeprazole	828	4673	0.18
3	Sulfamethoxazole	1478	6,499	0.23
4	Vancomycin	707	2,809	0.25
5	Phenobarbital	270	1,313	0.21
6	G-CSF	440	1,907	0.23
7	Acetaminophen	432	1,865	0.23
8	Nifedipine	327	1,411	0.23
9	Fusidine	286	1,183	0.24
10	Oseltamivir	270	1,070	0.25
11	Nystatin	459	1,722	0.27
12	Meropenem	795	2,712	0.29

*G-CSF, granu1ocyte colony-stimulating factor; DILI, drug-induced liver injury*.

a *Total number of drug users: we counted it only once although there was multiple administrations from admission to discharge*.

### Detection of DILI Signals

Table 2 listed the data extraction workflow of the above 12 suspect drugs. According to the design of stage 2, there were 2,004 cases in which fluconazole was used, along with 2,295 cases with omeprazole, 878 cases with sulfamethoxazole, 1,970 cases with vancomycin, 982 cases with phenobarbital, 2,051 cases with G-CSF, 1,609 cases with acetaminophen, 714 cases with nifedipine, 712 cases with fusidine, 957 cases with oseltamivir, 1,105 cases with nystatin, and 1,687 cases with meropenem in exposed groups. The exposed group and unexposed group were matched by gender, age, admission time, and major diagnosis with the ratio 1:4. The basic clinical information between two groups was described in Table S3.

**Table 2:** The data filtering workflow for 12 suspect drugs.

**Drug ID**	**Suspect drugs**	**Exposed group**					**Unexposed group**				
		**Exposed to suspect drug**	**At least 1 ALT or TB test before and after medication**	**Initial ALT or TB within ULN**	**Without hepatobiliary disease**	**Without hepatoprotectants**	**Not exposed to suspect drug**	**At least 2 ALT or TB tests**	**Initial ALT or TB test within ULN**	**Without hepatobiliary disease**	**Without hepatoprotectants**
1	Fluconazole	8,188	4,007	2,296	2,283	2,004	333,252	55,238	32,157	32,005	29,611
2	Omeprazole	24,408	4,339	2,686	2,654	2,295	317,032	53,054	30,605	30,494	28,134
3	Sulfamethoxazole	26,658	1,755	1,102	1,097	878	314,782	51,444	29,484	29,332	27,575
4	Vancomycin	10,855	3,813	2,367	2,354	1,970	330,585	55,058	31,817	31,963	29,418
5	Phenobarbital	7,071	1,794	1,080	1,077	982	334,369	57,570	33,580	33,419	30,661
6	G-CSF	23,698	4,481	2,539	2,529	2,051	317,742	53,813	31,185	31,033	29,027
7	Acetaminophen	9,224	2,822	1,838	1,828	1,609	332,216	56,485	32,742	32,587	30,028
8	Nifedipine	4,185	1,394	855	854	714	337,255	58,522	34,052	33,889	31,203
9	Fusidine	4,214	1,313	832	824	712	337,226	58,453	34,015	33,863	31,137
10	Oseltamivir	4,659	1,816	1,177	1,174	957	336,781	58,140	33,837	33,676	31,073
11	Nystatin	22,281	2,547	1,327	1,322	1,105	319,159	51,589	33,204	33,054	30,536
12	Meropenem	8,893	3,606	1,997	1,984	1,687	332,547	55,021	32,104	31,956	29,577

*ALT, alanine aminotransferase; G-CSF, granulocyte colony-stimulating factor; ULN, upper limit of normal range; TB, total bilirubin*.

**Figure 3:**
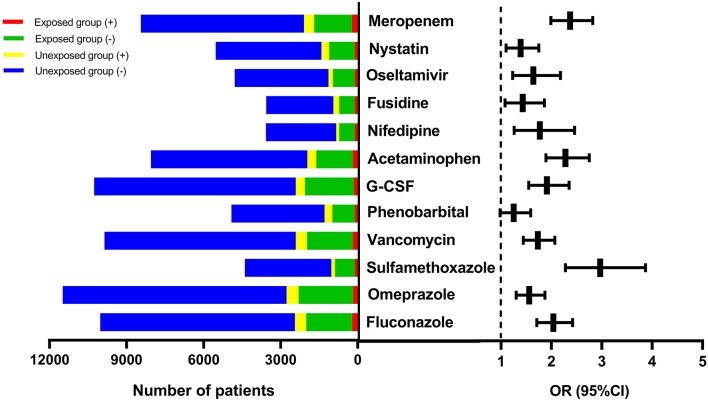
The forest plot for the 12 suspect drugs-DILI associations. DILI, drug-induced liver injury; OR, odds ratio; G-CSF, granulocyte colony-stimulating factor; CI, confidence interval.

Out of the 12 suspect drugs, fluconazole (OR = 2.04, 95%CI: 1.71–2.42, *P* < 0.0001), omeprazole (OR = 1.56, 95%CI: 1.30–1.87, *P* < 0.0001), sulfamethoxazole (OR = 2.97, 95%CI: 2.28–3.87, *P* < 0.0001), vancomycin (OR = 1.73, 95%CI: 1.44–2.07, *P* < 0.0001), G-CSF (OR = 1.91, 95%CI: 1.55–2.35, *P* < 0.0001), acetaminophen (OR = 2.28, 95%CI: 1.89–2.75, *P* < 0.0001), nifedipine (OR = 1.77, 95%CI: 1.26–2.46, *P* < 0.001), fusidine (OR = 1.43, 95%CI: 1.08–1.86, *P* = 0.01), oseltamivir (OR = 1.64, 95%CI: 1.23, 2.18, *P* < 0.001), nystatin (OR = 1.39, 95%CI: 1.10–1.75, *P* = 0.01), and meropenem (OR = 2.37, 95%CI: 1.99–2.82, *P* < 0.0001) were found to be associated with DILI as positive signals (shown in Figure 3). Although phenobarbital tended toward being a positive signal with regard to DILI, it did not reach statistically significance (OR = 1.25, 95%CI:0.98, 1.59, *P* = 0.068). Table 3 described the results of 12 drugs with regard to their associations with DILI.

**Table 3:** The result of DILI signal detection in a retrospective cohort study.

**Drug ID**	**Drugs name**	**Exposed group**		**Unexposed group**		**B**	***P***	**OR (95%CI)**
		**+**	**–**	**+**	**–**			
1	Fluconazole	212	1,792	439	7,577	0.71	<0.0001	2.04 (1.71, 2.42)
2	Omeprazole	177	2,118	471	8,709	0.45	<0.0001	1.56 (1.30, 1.87)
3	Sulfamethoxazole	105	773	151	3,361	1.09	<0.0001	2.97 (2.28, 3.87)
4	Vancomycin	187	1,783	444	7,436	0.55	<0.0001	1.73 (1.44, 2.07)
5	Phenobarbital	94	888	306	3,622	0.23	0.068	1.25 (0.98, 1.59)
6	G-CSF	144	1,907	363	7,841	0.65	<0.0001	1.91 (1.55, 2.35)
7	Acetaminophen	187	1,422	352	6,084	0.82	<0.0001	2.28 (1.89, 2.75)
8	Nifedipine	54	660	125	2,731	0.57	<0.001	1.77 (1.26, 2.46)
9	Fusidine	80	632	232	2,616	0.36	0.01	1.43 (1.08, 1.86)
10	Oseltamivir	72	885	179	3,649	0.5	<0.001	1.64 (1.23, 2.18)
11	Nystatin	105	1,000	307	4,113	0.33	0.01	1.39 (1.10, 1.75)
12	Meropenem	218	1,469	402	6,346	0.86	<0.0001	2.37 (1.99, 2.82)

*G-CSF, granu1ocyte colony-stimulating factor; OR, odds ratio; CI, confidence interval*.

### Evaluation of Observed DILI Signals

According to available knowledge at present, the novelty of 11 positive DILI signals observed in stage 2 were further evaluated (shown in Table 4). Two drugs, namely, nystatin and G-CSF, were found to be possible new DILI signals as they had not been previously documented in researches in pediatric population and adults. In addition, three other drugs, namely, nifedipine, fusidine and oseltamivir, have not been reported as being associated with liver injury in pediatric patients, although these associations have been found in adults. The remaining drugs have been reported as being associated with liver injury in both adults and pediatric individuals. In addition, for all drugs except nystatin, there were currently details regarding DILI as potentially ADEs in the SPCs.

**Table 4:** The novelty of the positive signals of DILI.

**Drug ID**	**Drugs**	**Literature (PUBMED)**^a^		**Literature (CNKI/Wanfang)**		**SPCs^b^**
		**Adults**	**Children**	**Adults**	**Children**	
1	Fluconazole	Yes	Yes	Yes	No	Yes^c^
2	Omeprazole	Yes	Yes	Yes	No	Yes
3	Sulfamethoxazole	Yes	Yes	Yes	Yes	Yes
4	Vancomycin	Yes	Yes	Yes	Yes	Yes
5	G-CSF	No	No	No	No	Yes
6	Acetaminophen	Yes	Yes	Yes	Yes	Yes
7	Nifedipine	Yes	No	Yes	No	Yes
8	Fusidine	Yes	No	Yes	No	Yes
9	Oseltamivir	Yes	No	Yes	No	Yes
10	Nystatin	No	No	No	No	No
11	Meropenem	Yes	Yes	Yes	Yes	Yes

a *Literature reviewed: (1) PUBMED: https://pubmed.ncbi.nlm.nih.gov; (2) Wanfang: http://www.wanfangdata.com.cn/index.html); (3) CNKI: https://www.cnki.net*.

b *SPCs reviewed: (1) Micromedex: https://www.ibm.com/watson-health/learn/micromedex); (2) FDA website: https://www.fda.gov/; (3) Drug instructions*.

c *Yes = drug–DILI association was documented*.

*DILI, drug–induced liver injury; G-CSF, granu1ocyte colony-stimulating factor; SPC, summary of product characteristics*.

## Discussion

We conducted a study on the development and application of a quantitative pharmacovigilance algorithm to identify signals of DILI from routine EHR data. This study used a two-stage designed algorithm with selecting offending medications firstly and then determining the associations between DILI and drugs. Two new DILI signals that have never been documented in pediatric population were found using the real world data from EHR. These may become candidate drugs for pharmacovigilance and causality assessment studies.

The association of nystatin with DILI was found to be a possible new signal. As far as we know, this has not previously been reported in published documents for patients of any age and was also not labeled in the SPCs. Nystatin is an antifungal agent widely used to treat oropharyngeal candidiasis, candidiasis of the skin, and cutaneous and mucocutaneous infections in pediatrics. The adverse effects listed in its SPCs include diarrhea, nausea, vomiting, abdominal pain, hypersensitivity reaction, and Stevens-Johnson syndrome. Unlike in the United States, nystatin tablets are still marketed for Chinese children and adolescents (>5 years old). Although liver injury has never been specifically mentioned in association with nystatin, it has been reported as an undesirable side effect of the use of other systemic antifungal agents. An *in vitro* study found that nystatin may decrease P-gp activity, indicating the possible mechanism of hepatotoxicity (17). A recent case report showed elevated liver enzymes after combining the cyclosporine and nystatin, due to drug interactions (18). Further investigations about the potential association between nystatin and hepatotoxicity are needed.

The association of G-CSF with liver injury can be considered another new signal. G-CSF is a blood modifying agent widely used to treat neutropenia in patients with non-myeloid malignancies, marrow transplantation, and acute myeloid leukemia treated with chemotherapy in pediatric patients. It can be used in children (except premature neonates, newborns and infants) with close monitoring in China. The adverse effects listed in the SPCs including rash, anemia, diarrhea, and bone pain. Although G-CSF has been on the market for many years, its liver safety in children is still unclear due to insufficient researches in specific population. Despite no report of such an association in pediatrics, several case reports have demonstrated that G-CSF may increase ALT or AST levels in adults (19). Our results are the first to show that G-CSF might be associated with adverse hepatic reactions in children, which needs further investigation.

Three other drug-DILI associations (nifedipine, fusidine and oseltamivir) were identified as potentially new signals in pediatrics. Nifedipine is a dihydropyridine calcium channel blocker that remains a commonly prescribed medication for hypertension in pediatric patients. A very rare but known drug adverse reaction of nifedipine is hepatotoxicity, which has been described in the literature in adults (20). Fusidine is widely used in treating severe staphylococcal infections in children. The hepatotoxicity of fusidine in adults, manifested as jaundice and abnormal liver function tests, has been reported in many adult studies. Oseltamivir is an ethyl ester prodrug used to prevent and treat infections caused by influenza A and B viruses. A report launched by Medicines and Healthcare Products Regulatory Agency (MHRA) showed that oseltamivir could induce DILI, without clearly indicating the patients' ages. In summary, although these drugs have been used worldwide, there are still some controversies regarding their hepatic safety in children due to the lack of evidence. Our study may provide more clues for further research in pediatrics.

Finally, the remaining 6 drug-DILI associations found in this study have been widely known in both the adult and pediatric populations based on the available descriptions in the SPCs and literature. This may suggest to some degree that our method can produce reliable results. On the other hand, some of the drugs implicated in DILI that were widely known were not found in our study, such as rifampicin, isoniazid, atorvastatin and so on. This result does not mean no such associations, but rather because the prevalence of drugs exposure was too limited to detect DILI signals in pediatric or in BCH hospital.

Data-driven analytic methods are a valuable aid to the detection of ADEs from large EHRs for drug safety monitoring (21). One of the most valuable methods is based on the traditional pharmacoepidemiological approach (22). The basic principle of these designs is to identify two groups of patients due to exposures or events retrospectively or prospectively and calculate the ratio of the drug-event associations (21). The cohort design provides more solutions for addressing putative confounders than the modified disproportionality analysis (DPA), which was originally developed on SRS (23, 24). Different designs based on this type of method, such as the new user cohort design, matched case-control designs and self-controlled designs, were determined to have the ability to track ADEs linked to medical products by many agencies. For instance, the Korean researchers have developed an approach, namely, Comparison of the Laboratory Extreme Abnormality Ratio (CLEAR), to identify possible ADE signals from abnormal value of laboratory test (11, 25, 26).

In the present study, our algorithm is a matched case-control design pharmacoepidemiological approach. In comparison with CLEAR, our 2-stage designed approach has certain advantages with regard to methodology. In the process of selecting the drugs suspected of causing DILI, we roughly assessed the potentialities by computing the ratio of ADEs to drug users. This important additional step increased the efficiency and speed of subsequent steps. In addition, more complicated confounders, such as relevant diagnoses with clear competing causes and medications that may affect the level of relevant laboratory indicators, were excluded to enhance the reliability and accuracy of the results. These final results suggest that our method is a valuable tool to facilitate earlier signal detection using routine EHR data.

Some limitations of this study should be considered. First, this study focuses on the detection of DILI signals using routine EHR database, whereas causality assessment was not involved. Large retrospective medical datasets have certain inherent difficulties for performing ADR causality assessments such as its incomplete data, uncontrollable confounding factors as well as difficulties in data extraction and algorithm execution. Next step we will prospectively collect data and use well-known causality assessment scales, such as Roussel Uclaf Causality Assessment Method (RUCAM), to verify the potential candidate drugs found in this study (15, 27). Second, some possible residual confounders, such as concomitant drugs, dose-related effects and the time-varying confounding by underlying diseases, were not excluded and could have led to potential bias or imprecision. Third, this study included only drugs with a large number of users for screening their possibility of causing DILI. This may lead a risk for missing potential drugs. We will mine the DILI signals for the remaining drugs in our next study.

Regulatory agencies have spared no efforts for facilitating ADE signal detection through multiple heterogeneous data sources at present (28–31). Notable progress has been made in China in establishing the project “China ADR Sentinel Surveillance Alliance” (CASSA). At present, we have developed an automated program based on this algorithm, and adapted to other ADEs besides DILI, such as drug-induced thrombocytopenia, neutropenia, anemia, and so on. In the next step, more attention will be paid to integrate these multiple modules to a drug safety monitoring platform to afford quick-response tools for pediatric clinicians and pharmacists. Future research will also focus on tighter integration of the structured data and clinical narratives in EHRs to improve the accuracy and scalability of the method.

## Conclusions

In this work, we demonstrated a pharmacovigilance method to explore potentially DILI signals using real word data. The two-stage designed algorithm was performed to select suspect drugs firstly and then determine the associations between DILI and drugs, respectively. We found that 11 drugs were possibly associated with hepatotoxicity, including two previously undocumented signals, three potentially new signals in children and six well-known signals. Our work promotes the application of EHR datasets in pharmacovigilance and offers candidate drugs for further causality assessment studies.

## Data Availability Statement

The raw data supporting the conclusions of this article will be made available by the authors, without undue reservation, to any qualified researcher.

## Ethics Statement

The study was reviewed and approved by the Institutional Ethics Committee of Beijing Children's Hospital in China (2019-k-5).

## Author Contributions

LJ and XW undertook work of framework design and overall guidance of whole research. YY, ZS, YX, XZ, ZD, RW, DF, and YL took responsibility for the data collection. YY and XN performed the data processing and statistical analysis. YY and LJ were responsible for the article writing and data interpretation. XP and QZ provided important methodological advice.

### Conflict of Interest

The authors declare that the research was conducted in the absence of any commercial or financial relationships that could be construed as a potential conflict of interest.
