# Supplementary material for: Signal Detection of Potentially Drug-Induced Liver Injury in Children Using Electronic Health Records
Source: Front Pediatr. 2020 Apr 16;8:171. doi: 10.3389/fped.2020.00171 (PMC7177017; doi:10.3389/fped.2020.00171)
Supplement: Supplementary file 1 [file Data_Sheet_1.docx]

Supplementary Material

# Supplementary Tables

**Table S1** The excluded hepatobiliary diseases with clear competing causes of liver injury

| ID | Category | ICD-10 Codes |
| --- | --- | --- |
| 1 | Hepatic and biliary tract neoplasm | C22.001, C22.201, D13.401, D13.502, C22.901, D37.603, Z51.103 |
| 2 | Diseases of the digestive system | K75.001, K75.902, K76.901, K76.904, K81.901, K82.201, K83.002, K83.003, K83.301, K83.502, K83.806, K85.101, K91.812, K91.8409 |
| 3 | Viral infections | B00.801+K77.0*, B00.802+K77.0*, B18.102, B18.106, B25.101+K77.0* |
| 4 | Genetic and metabolic disorders-related hepatopathy | E74.001, E80.603, E83.001, Q44.401, Q44.502, Q44.503, Q44.504, Q85.905 |
| 5 | Hepatomegaly with splenomegaly, not otherwise classified | R16.201, R16.101, R16.001, S36.102, S36.111 |

**Abbreviations:** ICD-10, international classification of diseases (Version 10).

**Table S2** The hepatoprotectants excluded in stage 2

| ID | Hepatoprotectants | Form |
| --- | --- | --- |
| 1 | Ornithine aspartate | Injection/Granules |
| 2 | Compound glycyrrhizin | Injection/Tablets |
| 3 | Ademetionine1,4-butanedisulfonate | Injection/Enteric Coated Tablets |
| 4 | Bifendate | Pills |
| 5 | Glucurolactone | Tablets |
| 6 | Reduced glutathione | Injection |
| 7 | Heparolysate | Injection |
| 8 | Hepatocyte growth-promoting factors | Injection |
| 9 | Ursodeoxycholic acid | Capsules |
| 10 | Sodium glucuronic acid | Injection |

**Table S3** The basic clinical information of the included records in exposed group and unexposed group

|  |  | Fluconazole | | Omeprazole | | Sulfamethoxazole | | Vancomycin | | Phenobarbital | | G-CSF | |
| --- | --- | --- | --- | --- | --- | --- | --- | --- | --- | --- | --- | --- | --- |
|  |  | EG | UEG | EG | UEG | EG | UEG | EG | UEG | EG | UEG | EG | UEG |
| Age |  | 3.51±3.84 | 3.33±3.45 | 5.83±4.28 | 5.68±4.35 | 6.11±4.01 | 5.99±4.21 | 4.14±3.71 | 4.24±3.66 | 2.68±3.60 | 2.58±3.30 | 5.02±3.50 | 5.06±4.02 |
|  | t | -1.99 | | -1.43 | | -0.77 | | 1.13 | | -0.81 | | 0.43 | |
|  | *P* | 0.05 | | 0.15 | | 0.44 | | 0.26 | | 0.42 | | 0.67 | |
| Gender(%) | Male | 1263  (63.02) | 5178  (64.60) | 1370  (59.69) | 5437  (59.23) | 535  (60.93) | 2229  (63.47) | 1211  (61.47) | 4890  (62.06) | 596  (60.69) | 2438  (62.07) | 1269  (61.87) | 5043  (61.47) |
|  | Female | 741  (36.98) | 2838  (35.40) | 925  (40.31) | 3743  (40.77) | 343  (39.07) | 1283  (36.53) | 759  (38.53) | 2990  (37.94) | 386  (39.31) | 1490  (39.93) | 782  (38.13) | 3161  (38.53) |
|  | χ^2^ | 1.73 | | 0.17 | | 1.93 | | 0.23 | | 0.63 | | 0.11 | |
|  | *P* | 0.19 | | 0.68 | | 0.16 | | 0.63 | | 0.43 | | 0.74 | |
| Major dignosis  (%) | Certain infectious and parastic diseases | 143  (7.14) | 541  (6.75) | 155  (6.75) | 685  (7.46) | 16  (1.82) | 61  (1.74) | 65  (3.30) | 238  (3.02) | 88  (8.96) | 369  (9.39) | 24  (1.17) | 81  (0.99) |
|  | Neoplasms | 254  (12.67) | 952  (11,88) | 234  (10.20) | 1058  (11.53) | 224  (25.51) | 847  (24.12) | 396  (20.10) | 1542  (19.57) | 53  (5.40) | 224  (5.70) | 336  (16.38) | 1003  (15.88) |
|  | Diseases of the blood and blood-forming organs involving the immune mechanism | 196  (9.78) | 746  (9.31) | 382  (16.64) | 1404  (15.29) | 111  (12.64) | 458  (13.04) | 217  (11.02) | 916  (11.62) | 50  (5.09) | 197  (5.02) | 740  (36.08) | 3048  (37.15) |
|  | Endocrine,nutritional and metabolic diseases | 13  (0.65) | 60  (0.75) | 23  (1.00) | 83  (0.90) | 2  (0.23) | 3  (0.09) | 7  (0.36) | 33  (0.42) | 9  (0.92) | 28  (0.71) | 6  (0.29) | 28  (0.34) |
|  | Mental and behavioural disorders | 0  (0.00) | 0  (0.00) | 0  (0.00) | 0  (0.00) | 0  (0.00) | 0  (0.00) | 0  (0.00) | 0  (0.00) | 5  (0.51) | 15  (0.38) | 0  (0.00) | 0  (0.00) |
|  | Diseases of the nervous system | 64  (3.19) | 258  (3.22) | 132  (5.75) | 539  (5.87) | 6  (0.68) | 20  (0.57) | 128  (6.50) | 513  (6.51) | 258  (26.27) | 1048  (26.68) | 3  (0.15) | 15  (0.18) |
|  | Diseases of the eye, adnex, ear and mastoid process | 2  (0.10) | 7  (0.09) | 3  (0.13) | 8  (0.09) | 0  (0.00) | 0  (0.00) | 1  (0.05) | 1  (0.01) | 2  (0.20) | 8  (0.20) | 0  (0.00) | 0  (0.00) |
|  | Diseases of the circulation system | 28  (1.40) | 85  (1.06) | 25  (1.09) | 70  (0.76) | 5  (0.57) | 16  (0.46) | 33  (1.68) | 93  (1.18) | 31  (3.16) | 126  (3.21) | 1  (0.05) | 2  (0.02) |
|  | Diseases of the respiratory system | 538  (26.85) | 2277  (28.41) | 237  (10.33) | 1047  (11.41) | 50  (5.69) | 219  (6.24) | 29  0(14.72) | 1181  (14.99) | 83  (8.45) | 388  (9.88) | 46  (2.24) | 150  (1.83) |
|  | Diseases of the digestsive system | 91  (4.45) | 361  (4.50) | 287  (12.51) | 1025  (11.17) | 7  (0.80) | 28  (0.80) | 50  (2.54) | 170  (2.16) | 16  (1.63) | 58  (1.48) | 12  (0.59) | 42  (0.51) |
|  | Diseases of the skin and suscutaneous tissue | 13  (.65) | 34  (0.42) | 18  (0.78) | 68  (0.74) | 1  (0.11) | 4  (0.11) | 22  (1.12) | 81  (1.03) | 1  (0.10) | 3  (0.08) | 5  (0.24) | 25  (0.30) |
|  | Diseases of the musculoskeletal system and connective tissue | 66  (3.29) | 296  (3.69) | 140  (6.10) | 492  (5.36) | 67  (7.63) | 322  (9.17) | 32  (1.62) | 135  (1.71) | 27  (2.75) | 112  (2.85) | 9  (0.44) | 39  (0.48) |
|  | Diseases of the genitourinary system | 33  (1.65) | 151  (1.88) | 69  (3.01) | 235  (2.56) | 18  (2.05) | 60  (1.71) | 0  (0.00) | 0  (0.00) | 28  (2.85) | 77  (1.96) | 2  (0.10) | 8  (0.10) |
|  | Certain conditions originating in the perinatal period | 58  (2.89) | 181  (2.26) | 1  (0.04) | 4  (0.04) | 0  (0.00) | 0  (0.00) | 11  (0.56) | 45  (0.57) | 32  (3.26) | 110  (2.80) | 0  (0.00) | 0  (0.00) |
|  | Congenital malformations, deformations and chromosomal abnormalities | 111  (5.54) | 465  (5.80) | 125  (5.45) | 604  (6.58) | 12  (1.37) | 4  7(1.34) | 68  (4.35) | 307  (3.90) | 191  (19.45) | 754  (19.20) | 10  (0.49) | 68  (0.83) |
|  | Symptoms, signs and abnormal clinical and laboratory findings, not elsewhere classified | 22  (1.10) | 57  (0.71) | 58  (2.53) | 254  (2.77) | 6  (0.68) | 17  (0.48) | 27  (1.37) | 91  (1.15) | 29  (2.95) | 122  (3.11) | 10  (0.49) | 70  (0.85) |
|  | injury,poisoning and certain other consequrences of external causes | 28  (1.40) | 117  (1.46) | 58  (2.53) | 213  (2.32) | 2  (0.23) | 3  (0.09) | 39  (1.98) | 172  (2.18) | 30  (3.05) | 105  (2.67) | 4  (0.20) | 50  (0.61) |
|  | External causes of morbididty and mortality | 0  (0.00) | 0  (0.00) | 0  (0.00) | 0  (0.00) | 0  (0.00) | 0  (0.00) | 0  (0.00) | 0  (0.00) | 0  (0.00) | 0  (0.00) | 0  (0.00) | 0  (0.00) |
|  | Factors influencing health status and contact with health services | 344  (17.17) | 1428  (17.81) | 348  (15.16) | 1391  (15.15) | 351  (39.98) | 1407  (40.06) | 584  (29.64) | 2362  (29.97) | 49  (4.99) | 184  (4.68) | 843  (41.10) | 3175  (38.70) |
|  | χ^2^ | 14.14 | | 21.74 | | 6.73 | | 8.52 | | 7.12 | | 16.5 | |
|  | *P* | 0.59 | | 0.15 | | 0.95 | | 0.90 | | 0.98 | | 0.28 | |
| Admission date time (year) (%) | 2010 | 222  (11.08) | 827  (10.32) | 149  (6.49) | 624  (6.80) | 105  (11.96) | 460  (13.10) | 206  (10.46) | 838  (10.63) | 146  (14.87) | 591  (15.30) | 198  (9.65) | 931  (11.35) |
|  | 2011 | 320  (15.97) | 1234  (15.39) | 225  (9.80) | 796  (8.67) | 102  (11.62) | 484  (13.78) | 226  (11.47) | 925  (11.74) | 141  (14.36) | 534  (13.85) | 171  (8.34) | 675  (8.23) |
|  | 2012 | 278  (13.87) | 1112  (13.87) | 266  (11.59) | 1079  (11.75) | 121  (13.78) | 460  (13.10) | 202  (10.25) | 815(10.34) | 103  (10.49) | 404  (10.29) | 228  (11.12) | 1035  (12.62) |
|  | 2013 | 189  (9.43) | 723  (9.02) | 305  (13.29) | 1287  (14.02) | 101  (11.50) | 440  (12.53) | 236  (11.98) | 920  (11.68) | 98  (9.98) | 426  (10.85) | 278  (13.55) | 1106  (13.48) |
|  | 2014 | 222  (11.08) | 907  (11.31) | 249  (10.85) | 1034  (11.26) | 124  (14.12) | 525  (14.95) | 251  (12.74) | 969  (12.30) | 108  (11.00) | 437  (11.13) | 230  (11.21) | 899  (10.96) |
|  | 2015 | 226  (11.28) | 944  (11.78) | 332  (14.47) | 1339  (14.59) | 122  (13.90) | 495  (14.09) | 254  (12.89) | 1008  (12.79) | 116  (11.81) | 457  (11.63) | 249  (12.14) | 933  (11.37) |
|  | 2016 | 248  (12.38) | 1038  (12.95) | 329  (14.34) | 1339  (14.59) | 78  (8.88) | 245  (6.98) | 282  (14.31) | 1132  (14.37) | 147  (14.97) | 557  (13.93) | 330  (16.58) | 1276  (15.55) |
|  | 2017 | 299  (14.92) | 1231  (15.36) | 440  (19.17) | 1682  (18.32) | 125  (14.24) | 403  (11.47) | 313  (15.89) | 1273  (16.15) | 123  (12.53) | 522  (13.03) | 367  (18.38) | 1349  (16.44) |
|  | χ^2^ | 2.56 | | 4.74 | | 12.29 | | 0.62 | | 1.62 | | 10.61 | |
|  | *P* | 0.92 | | 0.69 | | 0.09 | | 0.99 | | 0.98 | | 0.16 | |

**Abbreviations:** EG,exposed group; UEG, unexposed group; G-CSF, granu1ocyte colony-stimulating factor.

**Table S3** The basic clinical information of the included records in exposed group and unexposed group (Continued)

|  |  | Acetaminophen | | Nifedipine | | Fusidine | | Oseltamivir | | Nystatin | | Meropenem | |
| --- | --- | --- | --- | --- | --- | --- | --- | --- | --- | --- | --- | --- | --- |
|  |  | EG | UEG | EG | UEG | EG | UEG | EG | UEG | EG | UEG | EG | UEG |
| Age |  | 4.45±3.96 | 4.35±4.01 | 8.13±4.20 | 7.94±4.24 | 3.73±3.78 | 3.54±3.52 | 4.78±3.69 | 4.71±3.69 | 4.17±3.89 | 3.99±3.63 | 3.57±3.87 | 3.39±3.60 |
|  | t | -0.90 | | -1.00 | | -1.25 | | -0.53 | | -1.40 | | -1.83 | |
|  | *P* | 0.37 | | 0.30 | | 0.21 | | 0.59 | | 0.16 | | 0.07 | |
| Gender(%) | Male | 992  (61.65) | 4046  (62.87) | 411  (57.56) | 1706  (56.73) | 435  (61.10) | 1739  (61.06) | 576  (60.19) | 2342  (61.18) | 668  (60.45) | 2716  (61.45) | 1013  (60.04) | 4153  (61.54) |
|  | Female | 617  (38.35) | 2390  (37.13) | 303  (42.44) | 1150  (40.27) | 277  (38.90) | 1109  (38.94) | 381  (39.81) | 1486  (38.82) | 437  (39.55) | 1704  (38.55) | 674  (36.95) | 2595  (38.46) |
|  | χ^2^ | 0.81 | | 1.12 | | <0.001 | | 0.32 | | 0.37 | | 1.27 | |
|  | *P* | 0.37 | | 0.29 | | 0.99 | | 0.57 | | 0.54 | | 0.26 | |
| Dignosis(%) | Certain infectious and parastic diseases | 163  (10.13) | 629  (9.77) | 18  (2.52) | 64  (2.24) | 41  (5.76) | 12  6(4.42) | 20  (2.09) | 64  (1.67) | 57  (5.16) | 203  (4.59) | 103  (6.11) | 411  (6.09) |
|  | Neoplasms | 124  (7.71) | 483  (7.50) | 60  (8.40) | 255  (8.93) | 149  (20.93) | 564  (19.80) | 204  (21.32) | 731  (19.10) | 218  (19.73) | 789  (17.85) | 223  (13.22) | 848  (12.57) |
|  | Diseases of the blood and blood-forming organsinvolving the immune mechanism | 113  (7.02) | 466  (7.24) | 98  (13.73) | 410  (14.36) | 50  (7.02) | 197  (6.92) | 115  (12.02) | 432  (11.29) | 83  (7.51) | 323  (7.31) | 178  (10.55) | 642  (9.51) |
|  | Endocrine,nutritional and metabolic diseases | 11  (0.68) | 43  (0.67) | 18  (2.52) | 59  (2.07) | 4  (0.56) | 16  (0.56) | 4  (0.42) | 17  (0.44) | 22  (1.99) | 72  (1.63) | 9  (0.53) | 26  (0.39) |
|  | Mental and behavioural disorders | 3  (0.19) | 6  (0.09) | 0  (0.00) | 0  (0.00) | 0  (0.00) | 0  (0.00) | 0  (0.00) | 0  (0.00) | 2  (0.18) | 9  (0.20) | 0  (0.00) | 0  (0.00) |
|  | Diseases of the nervous system | 169  (10.50) | 719  (11.17) | 23  (3.22) | 96  (3.36) | 67  (9.41) | 301  (10.57) | 39  (4.08) | 147  (3.84) | 64  (5.79) | 259  (5.86) | 114  (6.76) | 494  (7.32) |
|  | Diseases of the eye, adnex, aear and mastoid process | 7  (0.44) | 17  (0.26) | 0  (0.00) | 0  (0.00) | 0  (0.00) | 0  (0.00) | 0  (0.00) | 0  (0.00) | 0  (0.00) | 0  (0.00) | 2  (0.12) | 10  (0.15) |
|  | Diseases of the circulation system | 37  (2.30) | 141  (2.19) | 21  (2.94) | 74  (2.59) | 7  (0.98) | 39  (1.37) | 9  (0.94) | 24  (0.63) | 10  (0.90) | 34  (0.77) | 22  (1.30) | 93  (1.38) |
|  | Diseases of the respiratory system | 378  (23.49) | 1618  (25.14) | 26  (3.64) | 87  (3.05) | 204  (28.65) | 879  (30.86) | 136  (14.21) | 575  (15.02) | 155  (14.03) | 745  (16.86) | 271  (16.06) | 1202  (17.81) |
|  | Diseases of the digestsive system | 48  (2.98) | 166  (2.58) | 6  (0.84) | 16  (0.58) | 18  (2.53) | 62  (2.18) | 7  (0.73) | 24  (0.63) | 26  (2.53) | 90  (2.04) | 118  (6.99) | 433  (6.42) |
|  | Diseases of the skin and suscutaneous tissue | 51  (3.17) | 186  (2.89) | 2  (0.28) | 6  (0.21) | 18  (2.53) | 78  (2.74) | 2  (0.21) | 6  (0.16) | 10  (0.90) | 43  (0.97) | 17  (1.01) | 66  (0.98) |
|  | Diseases of the musculoskeletal system and connective tissue | 220  (13.67) | 871  (13.53) | 194  (27.17) | 729  (25.53) | 27  (3.79) | 134  (4.71) | 45  (4.70) | 211  (5.51) | 57  (5.16) | 216  (4.89) | 104  (6.16) | 420  (6.22) |
|  | Diseases of the genitourinary system | 62  (3.85) | 252  (3.92) | 160  (22.41) | 685  (23.98) | 9  (1.26) | 34  (1.19) | 4  (0.42) | 9  (0.24) | 13  (1.18) | 46  (1.04) | 31  (1.84) | 96  (1.42) |
|  | Certain conditions originating in the perinatal period | 3  (0.19) | 8  (0.12) | 1  (0.14) | 8  (0.28) | 2  (0.28) | 2  (0.07) | 0  (0.00) | 0  (0.00) | 10  (0.90) | 43  (0.97) | 44  (2.61) | 153  (2.27) |
|  | Congenital malformations, deformations and chromosomal abnormalities | 58  (3.60) | 194  (3.01) | 6  (0.84) | 30  (1.05) | 5  (0.70) | 23  (0.81) | 13  (1.36) | 47  (1.23) | 13  (1.18) | 56  (1.27) | 123  (7.29) | 552  (8.18) |
|  | Symptoms, signs and abnormal clinical and laboratory findings, not elsewhere classified | 69  (4.29) | 254  (3.95) | 14  (1.96) | 44  (1.54) | 16  (2.25) | 54  (1.90) | 11  (1.15) | 25  (0.65) | 6  (0.54) | 19  (0.43) | 30  (1.78) | 84  (1.24) |
|  | injury,poisoning and certain other consequrences of external causes | 9  (0.56) | 33  (0.51) | 3  (0.42) | 16  (0.56) | 12  (1.69) | 45  (1.58) | 3  (0.31) | 13  (0.34) | 2  (0.18) | 2  (0.05) | 43  (2.55) | 168  (2.49) |
|  | External causes of morbididty and mortality | 0  (0.00) | 0  (0.00) | 1  (0.14) | 1  (0.04) | 0  (0.00) | 0  (0.00) | 0  (0.00) | 0  (0.00) | 1  (0.09) | 1  (0.02) | 0  (0.00) | 0  (0.00) |
|  | Factors influencing health status and contact with health services | 84  (5.22) | 350  (5.44) | 63  (8.82) | 276  (9.66) | 83  (11.66) | 294  (10.32) | 345  (36.05) | 1503  (39.26) | 356  (32.22) | 1470  (33.26) | 255  (15.12) | 1050  (15.56) |
|  | χ^2^ | 8.08 | | 7.14 | | 10.01 | | 11.41 | | 12.38 | | 13.05 | |
|  | *P* | 0.97 | | 0.97 | | 0.82 | | 0.65 | | 0.78 | | 0.67 | |
| Admission date time (year) (%) | 2010 | 261  (16.22) | 1015  (15.77) | 77  (10.78) | 283  (9.91) | 156  (21.91) | 626  (21.98) | 19  (1.99) | 65  (1.70) | 205  (18.55) | 758  (17.15) | 183  (10.85) | 709  (10.51) |
|  | 2011 | 316  (19.64) | 1219  (18.94) | 86  (12.04) | 314  (10.99) | 140  (19.66) | 529  (18.57) | 48  (5.02) | 183  (4.78) | 108  (9.77) | 458  (10.36) | 230  (13.63) | 926  (13.72) |
|  | 2012 | 160  (9.94) | 648  (10.07) | 96  (13.45) | 375  (13.13) | 96  (13.48) | 372  (13.06) | 68  (7.11) | 271  (7.08) | 132  (11.95) | 548  (12.40) | 181  (10.73) | 739  (10.95) |
|  | 2013 | 198  (12.31) | 822  (12.77) | 98  (13.73) | 418  (14.64) | 82  (11.52) | 341  (11.97) | 53  (5.54) | 228  (5.96) | 211  (19.10) | 830  (18.78) | 229  (13.57) | 922  (13.66) |
|  | 2014 | 146  (9.07) | 610  (9.48) | 76  (10.64) | 307  (10.75) | 36  (5.06) | 139  (4.88) | 91  (9.51) | 321  (8.39) | 155  (14.03) | 636  (14.39) | 184  (10.91) | 673  (9.97) |
|  | 2015 | 125  (7.77) | 484  (7.52) | 75  (10.50) | 292  (10.22) | 26  (3.65) | 109  (3.83) | 209  (21.84) | 880  (22.99) | 64  (5.79) | 228  (5.16) | 205  (12.15) | 819  (12.14) |
|  | 2016 | 211  (13.11) | 865  (13.44) | 103  (14.43) | 425  (14.88) | 110  (15.45) | 466  (16.36) | 191  (19.96) | 801  (20.92) | 150  (13.57) | 634  (14.34) | 263  (15.59) | 1074  (15.92) |
|  | 2017 | 192  (11.93) | 773  (12.01) | 103  (14.43) | 442  (15.48) | 66  (9.27) | 266  (9.34) | 278  (29.05) | 1079  (28.19) | 80  (7.24) | 328  (7.42) | 212  (12.57) | 886  (13.13) |
|  | χ^2^ | 1.17 | | 1.91 | | 0.92 | | 2.79 | | 2.65 | | 1.80 | |
|  | *P* | 0.99 | | 0.97 | | 0.99 | | 0.90 | | 0.92 | | 0.97 | |

**Abbreviations:** EG, exposed group; UEG, unexposed group.
